# Towards precision medicine: defining and characterizing adipose tissue dysfunction to identify early immunometabolic risk in symptom-free adults from the GEMM family study

**DOI:** 10.1080/21623945.2020.1743116

**Published:** 2020-04-09

**Authors:** Ernesto Rodriguez-Ayala, Esther C. Gallegos-Cabrales, Laura Gonzalez-Lopez, Hugo A. Laviada-Molina, Rocio A. Salinas-Osornio, Edna J. Nava-Gonzalez, Irene Leal-Berumen, Claudia Escudero-Lourdes, Fabiola Escalante-Araiza, Fatima A. Buenfil-Rello, Vanessa-Giselle Peschard, Antonio Laviada-Nagel, Eliud Silva, Rosa A. Veloz-Garza, Angelica Martinez-Hernandez, Francisco M. Barajas-Olmos, Fernanda Molina-Segui, Lucia Gonzalez-Ramirez, Rebeca Espadas-Olivera, Ricardo Lopez-Muñoz, Ruy D. Arjona-Villicaña, Victor M. Hernandez-Escalante, Martha E. Rodriguez-Arellano, Janeth F. Gaytan-Saucedo, Zoila Vaquera, Monica Acebo-Martinez, Judith Cornejo-Barrera, Huertas-Quintero Jancy Andrea, Juan Carlos Castillo-Pineda, Areli Murillo-Ramirez, Sara P. Diaz-Tena, Benigno Figueroa-Nuñez, Melesio E. Valencia-Rendon, Rafael Garzon-Zamora, Juan Manuel Viveros-Paredes, José Ángeles-Chimal, Jesús Santa-Olalla Tapia, José M. Remes-Troche, Salvador B. Valdovinos-Chavez, Eira E. Huerta-Avila, Juan Carlos Lopez-Alvarenga, Anthony G Comuzzie, Karin Haack, Xianlin Han, Lorena Orozco, Susan Weintraub, Jack W. Kent, Shelley A. Cole, Raul A. Bastarrachea

**Affiliations:** a Centro de Investigación en Ciencias de la Salud (CICSA), Facultad de Ciencias de la Salud, Universidad Anáhuac Norte, México City, México; b Facultad de Enfermería, Universidad Autónoma de Nuevo León (UANL), Monterrey, México; c Dirección de Postgrado e Investigación, Universidad del Valle de Atemajac (UNIVA), Zapopan, México; d Escuela de Ciencias de la Salud, Universidad Marista de Mérida, Yucatán, Mexico; e Facultad de Salud Pública y Nutrición (Faspyn), UANL, Monterrey, México; f Facultad de Medicina y Ciencias Biomédicas, Universidad Autónoma de Chihuahua, México; g Facultad de Ciencias Químicas, Universidad Autónoma de San Luis Potosí, México; h Population Health Program, Texas Biomedical Research Institute and Southwest National Primate Research Center (SNPRC), San Antonio, TX, USA; i Laboratorio de Inmunogenómica y Enfermedades Metabólicas, Instituto Nacional de Medicina Genómica, México City, México; j Laboratorio de Medicina Genómica del Hospital Regional Lic, Adolfo López Mateos, ISSSTE, Mexico City, Mexico; k Departamento de Enseñanza, Postgrado e Investigación, Hospital Infantil de Tamaulipas, Ciudad, México; l Departamento de Nutrición Humana, Universidad Latina de América, Morelia, México; m Clínica de Enfermedades Crónicas y Procedimientos Especiales (CECYPE), Morelia, México; n Facultad de Medicina, Universidad Autónoma Estado de Morelos, Cuernavaca, México; o Instituto de Investigaciones Médico-Biológicas, Universidad Veracruzana, Veracruz, México; p School of Medicine & South Texas Diabetes and Obesity Institute, University of Texas Rio Grande Valley, Edinburg, TX, USA; q The Obesity Society, Silver Spring Maryland, USA; r Department of Medicine, Sam and Ann Barshop Institute for Longevity and Aging Studies, University of Texas Health San Antonio, San Antonio, TX, USA; s Department of Biochemistry, University of Texas Health Science Center, San Antonio, TX, USA

**Keywords:** Adipose tissue dysfunction, immunometabolism, postprandial tissue biopsies, non-coding microRNAs, shotgun lipidomics

## Abstract

Interactions between macrophages and adipocytes are early molecular factors influencing adipose tissue (AT) dysfunction, resulting in high leptin, low adiponectin circulating levels and low-grade metaflammation, leading to insulin resistance (IR) with increased cardiovascular risk. We report the characterization of AT dysfunction through measurements of the adiponectin/leptin ratio (ALR), the adipo-insulin resistance index (Adipo-IRi), fasting/postprandial (F/P) immunometabolic phenotyping and direct F/P differential gene expression in AT biopsies obtained from symptom-free adults from the GEMM family study. AT dysfunction was evaluated through associations of the ALR with F/P insulin-glucose axis, lipid-lipoprotein metabolism, and inflammatory markers. A relevant pattern of negative associations between decreased ALR and markers of systemic low-grade metaflammation, HOMA, and postprandial cardiovascular risk hyperinsulinemic, triglyceride and GLP-1 curves was found. We also analysed their plasma non-coding microRNAs and shotgun lipidomics profiles finding trends that may reflect a pattern of adipose tissue dysfunction in the fed and fasted state. Direct gene differential expression data showed initial patterns of AT molecular signatures of key immunometabolic genes involved in AT expansion, angiogenic remodelling and immune cell migration. These data reinforce the central, early role of AT dysfunction at the molecular and systemic level in the pathogenesis of IR and immunometabolic disorders.

## Introduction

AT dysfunction plays a key role and is at the core of IR development [1]. IR is also a chronic systemic inflammatory syndrome primarily triggered by macrophage infiltration into adipose tissue [2]. Interactions between macrophages and adipocytes [3] are the early molecular factors influencing AT dysfunction, resulting in local altered adipose tissue metabolism that then progresses to altered systemic metabolism (mainly high leptin and low adiponectin circulating levels) and chronic low-grade inflammation, ultimately leading to IR [4]. IR, the hallmark of immunometabolic disorders [5], contributes to glucotoxicity, lipotoxicity, metaflammation, endothelial dysfunction, increased cardiovascular disease (CVD) risk and atherosclerosis [6].

The recently developed adiponectin/leptin ratio (ALR) correlates with IR better than adiponectin or leptin alone. It is strongly associated with surrogate measures of IR: the homoeostatic model assessment (HOMA) and the hyperinsulinemic-euglycemic clamp [7]. This emerging index decreases with increasing number of cardiometabolic risk factors reﬂecting the functionality of adipose tissue and negatively correlates with markers of low-grade chronic subclinical inﬂammation. It has been proposed that an ALR ≥ 1.0 can be considered normal, a ratio between ≥0.5 and <1.0 suggests moderate-medium increased risk, and a low ratio <0.5 indicates a severe increase in cardiovascular and immunometabolic risk [8]. A low (L)ALR as a marker of adipose tissue (AT) dysfunction is characterized by a lower secretion of adiponectin in relation to leptin levels, unhealthy adipose tissue hypoxia, proinﬂammatory macrophage polarization, altered adipokine proﬁle and IR [9].

The most relevant age-related chronic immunometabolic diseases (type 2 diabetes [T2D] and CVD) associated with increased mortality are slowly progressive, associated with traditional CVD risk factors [10] and with a symptom-free onset [5]. These pathologies are the leading cause of death around the world [11]. Cardiovascular risk phenotypes of immunometabolic origin (CVRIMO) – IR, hyperinsulinemia, dysglycemia, and dyslipidemia, elevated hsCRP and fibrinogen – are considered major risk factors for T2D and CVD [6]. The central underlying mechanisms triggering these F/P CVRIMO relate to localized immunometabolic processes at a cellular level during AT expansion (hypoxia, inflammation, inappropriate extracellular matrix (ECM) remodelling, impaired angiogenesis, fibrosis) defined as AT dysfunction [4].

Although fasting and postprandial dysglycemia, lipid-lipoprotein abnormalities, excess of body fat, and elevated systolic/diastolic blood pressure [10] are well-established traditional cardiometabolic risk factors [12], there is growing evidence to suggest that a metabolic inflammatory state, termed metaflammation [13] and defined as low-grade, chronic subclinical inflammation [5] orchestrated by metabolic cells in response to excess nutrients and energy [14] may be the underlying mechanism that determines whether or not an individual would develop these CVRIMO and cardiovascular risk abnormalities [15]. They are primarily associated with IR and an unfavourable inflammatory state characterized by high circulating levels of high-sensitivity C-reactive protein (hs-CRP), alpha necrosis tumour factor (TNF-α), hyperfibrinogenemia [16] and interleukin 6 (IL-6) [17].

The GEMM family study (Genética de las Enfermedades Metabólicas en México, Genetics of Metabolic Diseases in Mexico) is a bi-national, multi-centre collaborative study of CVRIMO related to T2D and the risk of cardiovascular and immunometabolic disease. GEMM’s study design characterizes detailed dynamic and function-based metabolic and molecular phenotypes in fasting and fed states (including the phenome, transcriptome, proteome and metabolome) in symptom-free volunteers. Data are acquired from the circulation and from F/P adipose tissue and skeletal muscle biopsies, key tissues to understand F/P metaflammation, insulin action and carbohydrate/lipid homoeostasis. All measurements in blood are taken over a time course of 5 h to allow fine-scale profiling of individual postprandial responses [18].

The aim of this paper is to introduce the methodology to characterize early, key contributors triggering the pathogenesis of AT dysfunction with data collected from the GEMM cohort. We studied the immunometabolic characteristics in the symptom-free female volunteers and compared the relation and trends of the high vs. low ALR with their F/P insulin-glucose axis, lipid-lipoprotein metabolism, and systemic inflammatory markers. In addition, we analysed whether their plasma non-coding exosomal [19] microRNAs (miRNAs) [20], advanced plasma shotgun lipidomics profiles [21], and particularly, direct adipose tissue gene expression, could differentially display molecular patterns reflecting AT dysfunction in the fed and fasted state.

## Materials and methods

### Subjects

The GEMM study involves an ongoing recruitment from 10 University-based metabolic research units and their affiliated teaching hospitals across Mexico, as described previously [18]. The goal of our broader, current (longitudinal), ongoing recruitment is to randomly recruit 400 symptom-free adult volunteers in ~10 extended families. Although this manuscript is only presenting data on females, GEMM has performed the 5 h meal challenge and biopsies of subcutaneous adipose tissue and skeletal muscle sample collection for 125 symptom-free participants until date (80 females and 45 males) [18]. A registered nurse performed a complete family and personal medical history on each participant that included information about allergies, illnesses, surgeries, immunizations, results of physical exams, tests, and screenings. It accurately identified people with a higher-than-usual chance of having common disorders, such as heart disease, high blood pressure, stroke, certain cancers, and diabetes. A second questionnaire was also performed to gather data on physical activity and food intake. It is a reliable and sensitive instrument that has been assessed and used in Mexican population [22]. Exclusion criteria include women that were pregnant or attempting to become pregnant, individuals with acute illness, activity-limiting unexplained illness, hypertension, dyslipidemia, prevalent diabetes, known cardiovascular or chronic lung disease, cancer or renal failure [23]. Individuals with signs of infection were also excluded. Participants were also ruled out for any evidence of likely atherosclerotic disease or risk by questioning them if they ever had coronary artery disease, a heart attack, or congestive heart failure [23]. Local ethics committee approval was obtained at each recruiting centre. Subjects were given a written and an oral explanation of the study, and all provided informed consent. Export of GEMM samples for multiOMICS analysis to the U.S. (Texas Biomedical Research Institute, San Antonio, TX), has been permitted by the Mexican Federal government in accordance with Mexican Genetic Sovereignty Law [24] (COFEPRIS Permit No. COF187278 (DEAPE 133300CT190038/2013) issued on 19 March 2013) [25].

### Study design

**Figure 1.**
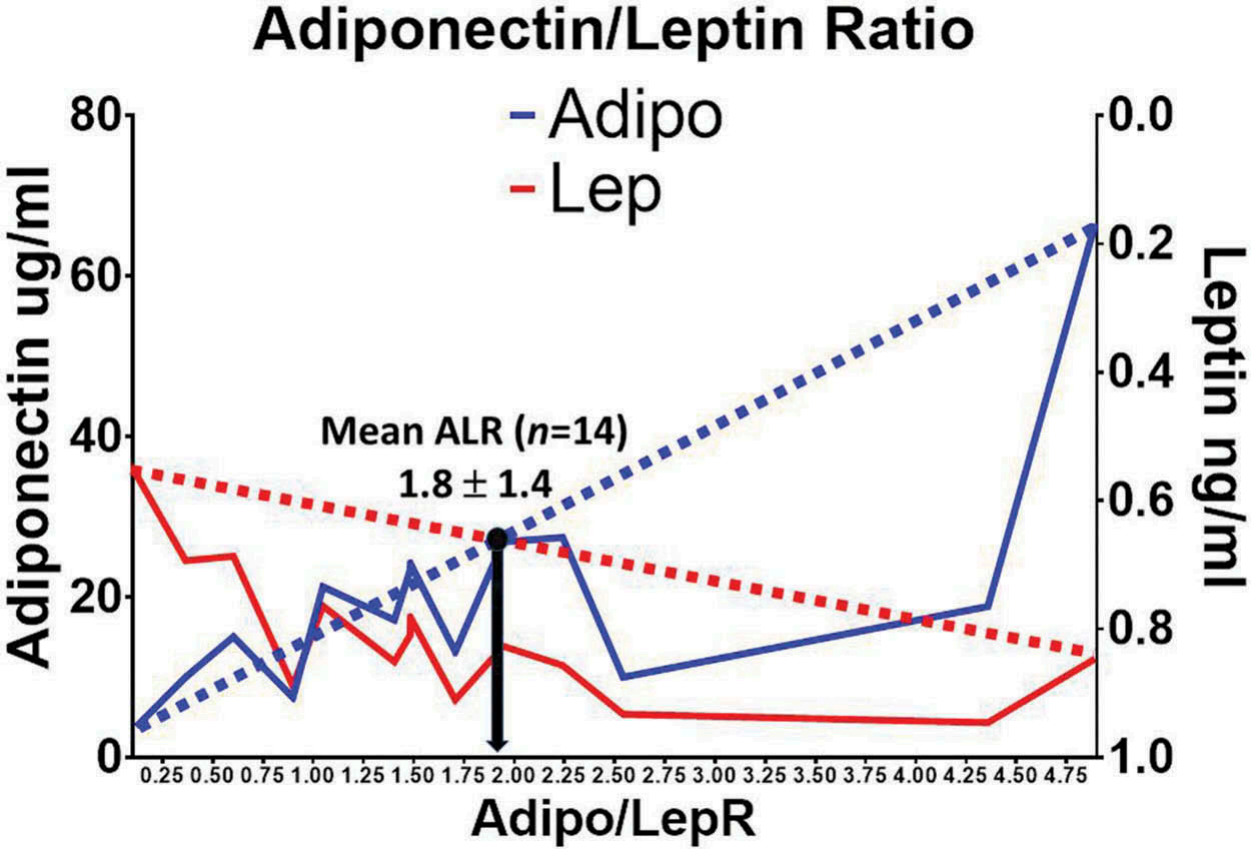
Mean ALR in our symptom-free female participants

Limited funds were obtained from a research granting foundation awarded to conduct a cross-sectional analysis from 14 symptom-free female adults chosen from the total female cohort (*n* = 80) to determine AT dysfunction at a systemic and at a molecular level (Table 1). We used the adiponectin and leptin circulating measures as phenotypes to determine the adiponectin/leptin ratio (ALR) trait on our 14 female participants (Figure 1). It allowed us to obtain an accurate mean to determine high (H) or low (L) ALR ratios among our chosen females [8]. We compared their mean (H) and (L) ALR to screen for trends of presence or absence of cardiovascular and immunometabolic early risk related to their AT dysfunction. Measured fasting and 2 h postprandial blood glucose, haemoglobin A1 c and insulin were used to rule out participants with evidence of T2D, prediabetes or metabolic syndrome (triglycerides ≥ 150 mg/dL, HDL cholesterol <40 mg/dL in men and <50 mg/dL in women or participants currently taking prescribed medicine for high cholesterol, blood pressure ≥130/85 mmHg or any participant currently taking prescription for hypertension) [23]. The presence of likely non-alcoholic fatty liver disease (NAFLD) or non-alcoholic steatohepatitis (NASH) was also ruled out by finding out whether a participant did not have alcohol dependence or laboratory data suggesting likely NASH [26].

**Table 1.** Comparison of demographic and biochemical characteristics between the *n* = 80 vs. *n* = 14 GEMM symptom-free female individuals, showing strong anthropometric and metabolic similarities and non-significant differences

Females	*n* = 80	*n* = 14	
Demographic characteristics and metabolic parameters	Mean ± SD	Mean ± SD	*P*
Age	38.8 ± 13.34	36.39 ± 12.11	0.499
Weight (kg)	70.48 ± 15.92	67.64 ± 13.31	0.361
Waist Circunference (cm)	88.43 ± 14.24	87.46 ± 13.06	0.819
BMI (kg/m^2^)	28.17 ± 5.84	28.95 ± 5.78	0.642
% Fat Total	38.58 ± 10.39	43.9 ± 5.43	0.053
Systolic Pressure (mmHg)	107.16 ± 10.48	108.14 ± 10.48	0.848
Diastolic Pressure (mmHg)	68.88 ± 8.14	69.0 ± 6.64	0.885
Glucose (mg/dL)	93.49 ± 22.92	86.71 ± 7.18	0.125
Triglycerides (mg/dL)	117.73 ± 56.77	128.78 ± 56.77	0.258
Total cholesterol (md/dL)	164.43 ± 43.09	165.07 ± 39.30	0.703
HDL- cholesterol (md/dL)	47.08 ± 14.75	43.71 ± 9.07	0.612
LDL- cholesterol (md/dL)	94.51 ± 34.22	98.71 ± 32.08	0.605

### Meal challenge, tissue biopsies and postprandial sample collection

GEMM participants were given an innovative balanced mixed meal challenge containing a healthy combination of carbohydrates, fats, and protein, dosed at 30% of each participant’s daily resting energy expenditure in Kcal (macronutrient composition: 65% carbohydrate, 15% protein and 20% fat). They also provided blood samples at defined timepoints over a 5 h time course, as well as subcutaneous adipose tissue biopsies under local anaesthesia at fasting and 180 min postprandium to represent fasting and peak of postprandial response. The fasting biopsies were taken after a two-week interval rather than on the same day to avoid post-surgical inflammatory effects on the postprandial gene and protein expression as it could happen if both biopsies are taken the same day [18].

### Postprandial biochemical phenotyping

Biochemical phenotypes were analysed on a Luminex 100IS platform (SBH Sciences, Natick, MA, USA) and an Immulite 1000 (Diamond Diagnostics Inc., Holliston, MA, USA) which run enzyme-linked immunosorbent assay (ELISA) and radioimmunoassay (RIA) analyses, consisting of an advanced optometric flux designed to analyse different cytokines and chemokines in less than 2 h in ~25 μL of plasma. We measured and analysed a wide range of clinical biochemistries (including liver function enzymes), hormones, cytokines and endophenotypes that define the fasting and postprandial cardiovascular and immunometabolic status of the study participants relative to inflammation, insulin resistance, and risk of CVD and T2DM. These analyte categories include: Beta cell and insulin-glucose axis: F/P insulin, GLP-1 and glucose curves, HOMA-IR; Adipose tissue function: leptin, adiponectin, the adipo-insulin resistance index (Adipo-IRi); Inflammation endophenotypes: hsCRP, PAI-1, TNF-α, IL-6, MCP-1, IL-10; Lipid-lipoprotein metabolism: non-esterified fatty acids (NEFA), F/P triglyceride curves and total cholesterol.

### Fasting and postprandial plasma shotgun lipidomics

Lipids in biological samples were extracted with chloroform/methanol in the presence of a cocktail of internal standards (at least one for each lipid class) using a modification of the method of Bligh and Dyer [27], and analysed by multi-dimensional mass spectrometry-based shotgun lipidomics (MDMS-SL), as previously described [28]. MDMS-SL determines all of the building blocks of individual species by using the combined analyses of multiple neutral loss scans (NLS) and/or precursor-ion scans (PIS) and processing the NLS/PIS data with software developed to identify the individual species [29]. Quantification of identified lipid species was automatically achieved through the use of this software for comparison of either survey mass spectra or NLS/PIS spectra to the internal standard of the corresponding lipid class [29]. This strategy permitted identification and quantification of hundreds to thousands of individual lipid species in nearly 50 lipid classes, including the following: triglycerides, diglycerides, monoglycerides, cholesterol and cholesteryl esters, oxysterols, acylcarnitines, acyl-CoAs, phospholipids (including cardiolipin), lysophospholipids, eicosanoids, 4-hydroxy alkenals and retinoic acid [30].

### miRNA sequencing

For miRNA Sequencing of plasma exosomes, miRNA was isolated from 200 ul plasma using the miRNeasy Serum/Plasma Advanced Kit (Qiagen) according to the manufacturer protocol. Quality and quantity were assessed by Agilent HS RNA assay and Qubit microRNA assay, respectively. Conversion of miRNA into cDNA library was performed using the NEXTflex Illumina Small RNA-Seq Kit v3 (BiooScientific). miRNA is heat denatured to allow ligation at the 3ʹ then 5ʹ ends with adapters that contain 4 bp randomized end sequences, and undergo a first and second strand cDNA synthesis resulting in dscDNA with blunt ends. Adapter ligation products were purified and enriched by PCR to create cDNA libraries. Gel-free size selection was performed to remove adapter-dimer product in the final cDNA library. Library quality and quantity were assessed by Agilent D1000 assay and Qubit dsDNA assay, respectively. Forty-eight sample libraries were pooled and then quantified by qPCR using the Kapa Library ABI Prism Quantification kit. Library pools were shipped out for sequencing. The sequence reads were aligned and quantified at the Texas Biomedical Research Institute in San Antonio, TX, [Molecular Services Core (MSC)] using an established pipeline in Partek Flow.

### RNA extraction, amplification and labelling from subcutaneous adipose tissue biopsies

Total RNA was isolated from adipose tissue using the RiboPure kit (Applied Biosystems) after homogenizing the RNALater-stabilized tissues in TRI Reagent. The quantity and quality of the RNA samples were determined using a NanoDrop ND-1000 spectrophotometer. Samples whose A260/A280 ratio deviate ±0.2 from the accepted ratio of 2.0 were excluded. The quality/integrity of the RNA was assessed using the Agilent RNA 6000 Nano LabChip Kit and an Agilent 2100 Bioanalyzer (Agilent Technologies), ensuring that the 28 S and 18 s ribosomal RNA species are intact and that significant degradation had not occurred. The concentration of the resulting RNA samples was determined by using the NanaoDrop ND-1000 spectrophotometer. For synthesis of cRNA, the Illumina TotalPrep RNA Amplification Kit (Applied Biosystems) was used. The quality of the cRNA was assessed using the Agilent RNA 6000 Nano LabChip Kit and an Agilent 2100 Bioanalyzer (Agilent Technologies). cRNA quantity was measured using a NanoDrop ND-1000 spectrophotometer.

### Subcutaneous fat gene expression profiling

We used commercially available Illumina *Human WG-6 y3.0* Expression BeadChips for whole genome expression analysis. These BeadChips contain six arrays, each with >48,000 probes. Each array provides genome-wide transcriptional coverage of well-characterized genes, gene candidates and splice variants. About 1.8 million beads are available to quantify mRNA levels for each sample providing on average a 30x redundancy and therefore a very high precision of detection. This system uses a ‘direct hybridization’ approach, whereby gene-specific probes attached to beads on the array are used to capture and detect labelled cRNAs. After hybridization, detection of cRNAs was achieved by using an Illumina BeadArray™ reader. Gene expression data were generated using the BeadStudio software package. This application reports quality of performance based on built-in controls that accompany each experiment. The resulting ~48,000 quantitative measures were entered into a phenotypic database and additional statistical quality control procedures were performed to prepare these phenotypes for statistical analysis.

### Statistical analysis data

are presented as mean ± standard deviation (SD) unless otherwise indicated. Differences between groups were analysed by two-tailed unpaired Student’s t-tests, as appropriate. The calculations were performed using SPSS23 (SPSS, Chicago, IL, USA) and GraphPad Prism 6 (GraphPad Software, Inc., La Jolla, CA, USA). Scatter plot mathematical diagrams using Cartesian coordinates to display two-dimensional data visualization using dots were applied to represent association between two different variables.

## Results

**Figure 2.**
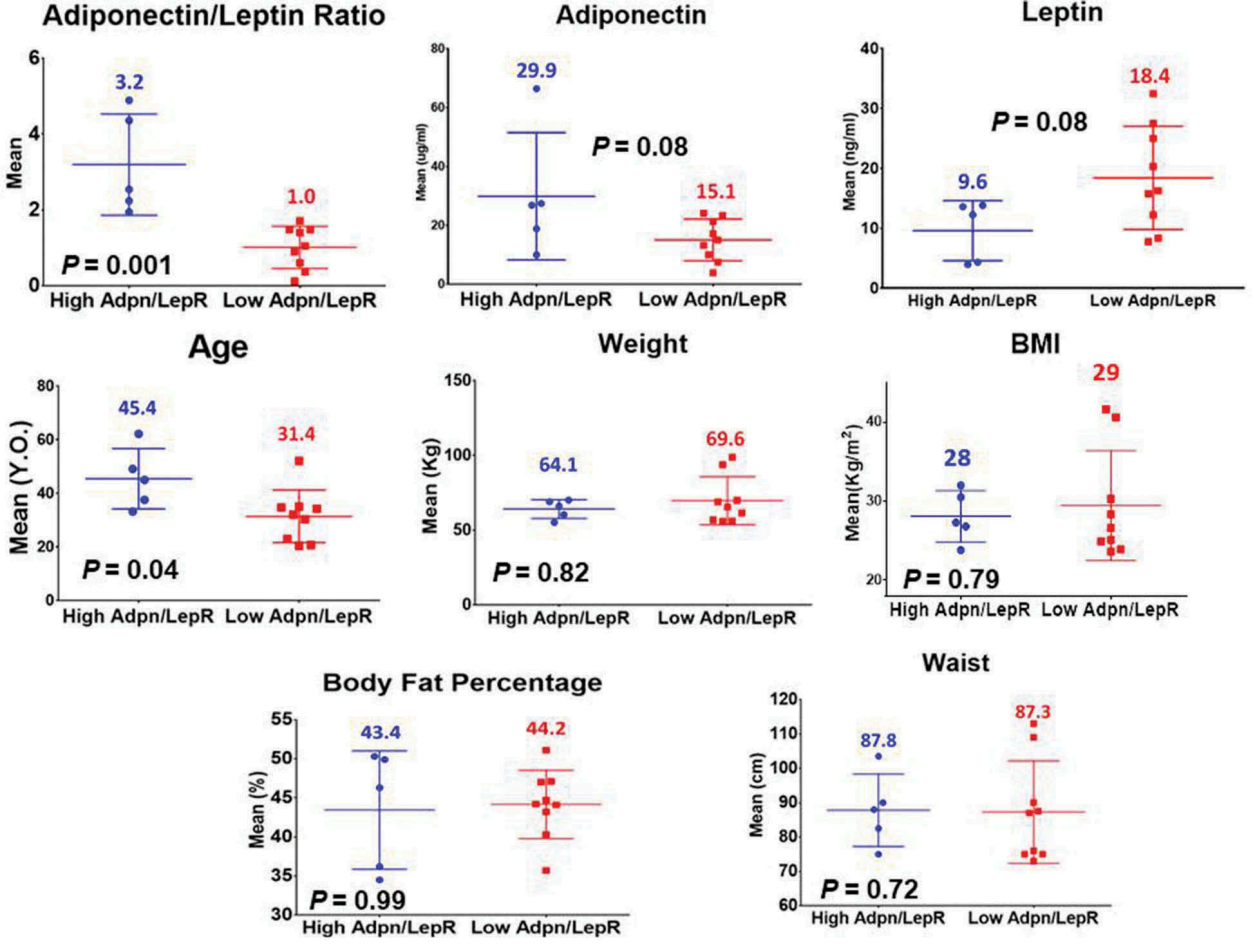
Mean leptin, adiponectin, Adiponectin/Leptin ratio and anthropometric phenotypes of participants in the GEMM study (ALR and Age with a *P-*value ≤ 0.05)

Table 1 shows the demographic characteristics and metabolic parameters of the 80 female GEMM volunteers including age, weight, height, BMI, fasting glucose, total cholesterol, HDL cholesterol, triglycerides, and blood pressure (systolic and diastolic). Deep phenotyping (HOMA-IR, postprandial metabolism curves, F/P plasma microRNA signatures, shotgun F/P lipidomics, profiling of inflammatory markers [31] and F/P adipose tissue transcriptomics) on 14 of our GEMM participants is also shown in Table 1. They were chosen for deep phenotyping because their clinical characteristics show similar metabolic patterns that accurately represent the full database of 80 women. Figure 1 shows a mean Adiponectin/Leptin ratio (ALR) of 1.8 ± 1.4 in 14 of our symptom-free participants. We decided to designate the females with an ALR above and below the mean of 1.8 as having a high (H) and a low (L) ALR. Although this arbitrary cut-off point is above the limits of a healthy ALR (>1.0) [8], the subgroup of females with an ALR above and below the mean 1.8 fits our hypothesis that adipose tissue dysfunction can be documented among symptom-free individuals. Figure 2 and Table 2 show that our symptom-free subjects had a mean (L) (1.0 **±** 0.56) and (H) (3.2 **± **1.3) ALR. Mean plasma concentrations of total fasting adiponectin and leptin were 29.9 ± 21.6 ug/mL and 9.6 ± 5.0 ng/mL in the (H)ALR group, and 15.1 ± 7.12 ug/mL and 18.4 ± 8.6 ng/mL in the (L)ALR, respectively.

**Table 2.** High (H: >1.8) and Low (L: <1.8) Adiponectin/Leptin ratio (ALR) according to adipokine concentrations in symptom-free female volunteers who underwent deep phenotyping. *P*-values for adiponectin mean levels: 0.08; leptin mean levels: 0.08; ARL: 0.001. (A p-value ≤ 0.05 is statistically significant)

Symptom-free female adults (*N* = 14)	Fasting Adiponectin Levels (ug/ml)	ADPN/LEP RATIO	Fasting Leptin Levels (ng/ml)
MTY0017	66.5	4.89	13.6
MTY0003	18.9	4.36	4.3
MTY0007	10.0	2.54	3.9
MTY0006	27.4	2.24	12.2
MTY0014	26.9	1.95	13.8
MEAN (H) ALR	29.9	3.2	9.6
MTY0013	13.2	1.71	7.7
MTY0020	24.1	1.48	16.3
MTY0018	23.4	1.48	15.8
MTY0019	17.2	1.40	12.2
MTY0021	21.3	1.05	20.3
MTY0009	7.5	0.90	8.4
MTY0015	15.1	0.60	25.0
MTY0016	10.0	0.36	27.5
MTY0010	3.9	0.12	32.4
MEAN (L) ALR	15.1	1.0	18.4

Mean values in (H) vs. (L)ALR for weight, BMI, waist circumference and body fat percentage showed that these anthropometric measurements were higher in the (L)ALR group. Age showed that the (L)ALR group was younger (Figure 2). Most mean values did not reach statistical significance.

**Figure 3.**
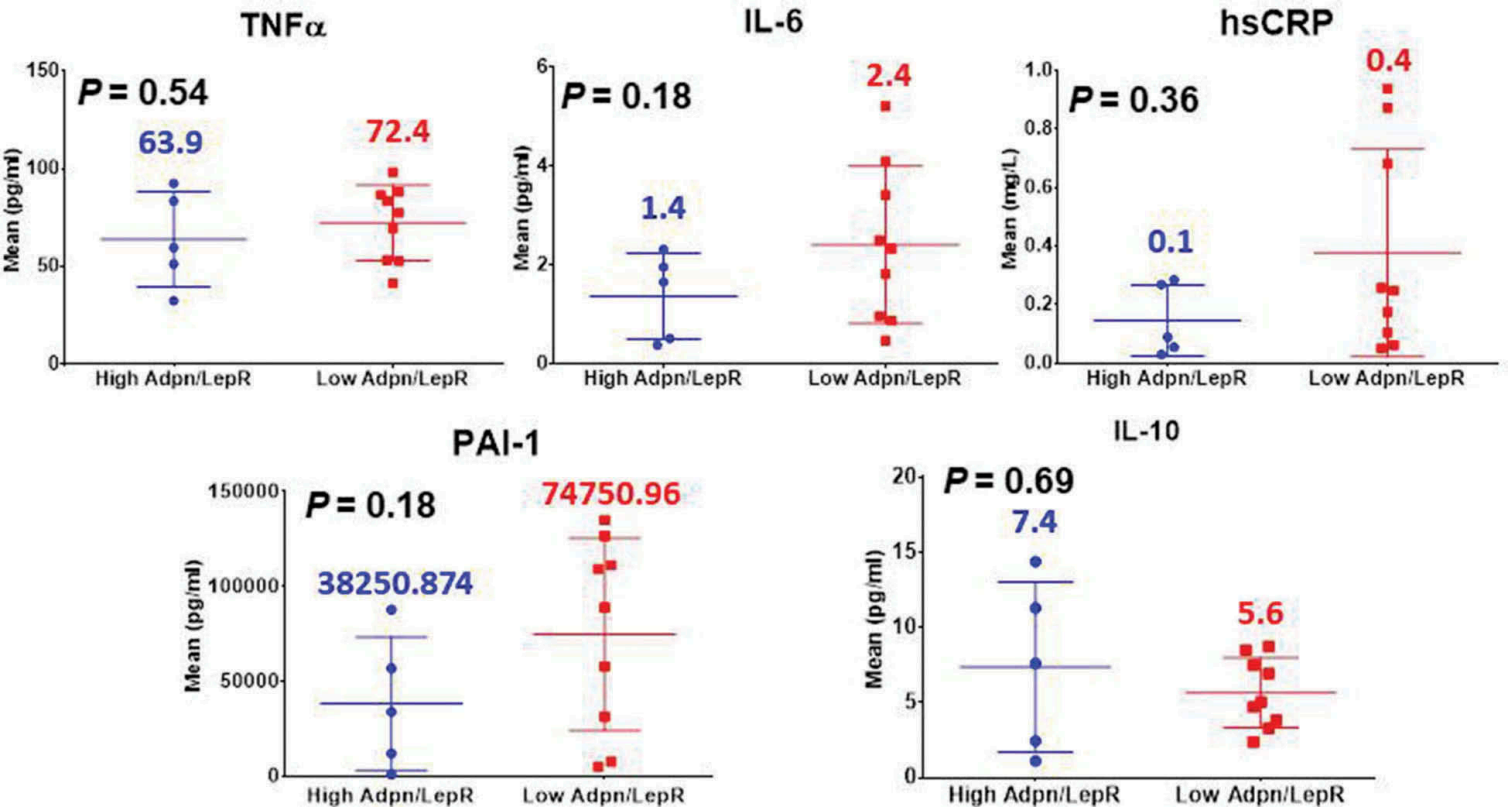
Immunometabolic and proinflammatory profile of participants in the GEMM study (non-significant *P*-values)

Proinflammatory fasting systemic biomarker levels between the (H) and (L)ALR groups are shown in Figure 3. Our preliminary data showed higher systemic levels of TNF-α in the females with (L)ALR. Circulating levels of IL-6 were elevated among the symptom-free volunteers with (L)ALR. Our results showed a trend of elevated systemic hs-CRP among subjects with (L)ALR. The pattern for circulating levels of PAI-1 in the subgroup with (L)ALR showed a relevant elevation. IL-10 was decreased in the (L)ALR group. However, the *P*-values were statistically non-significant.

**Figure 4.**
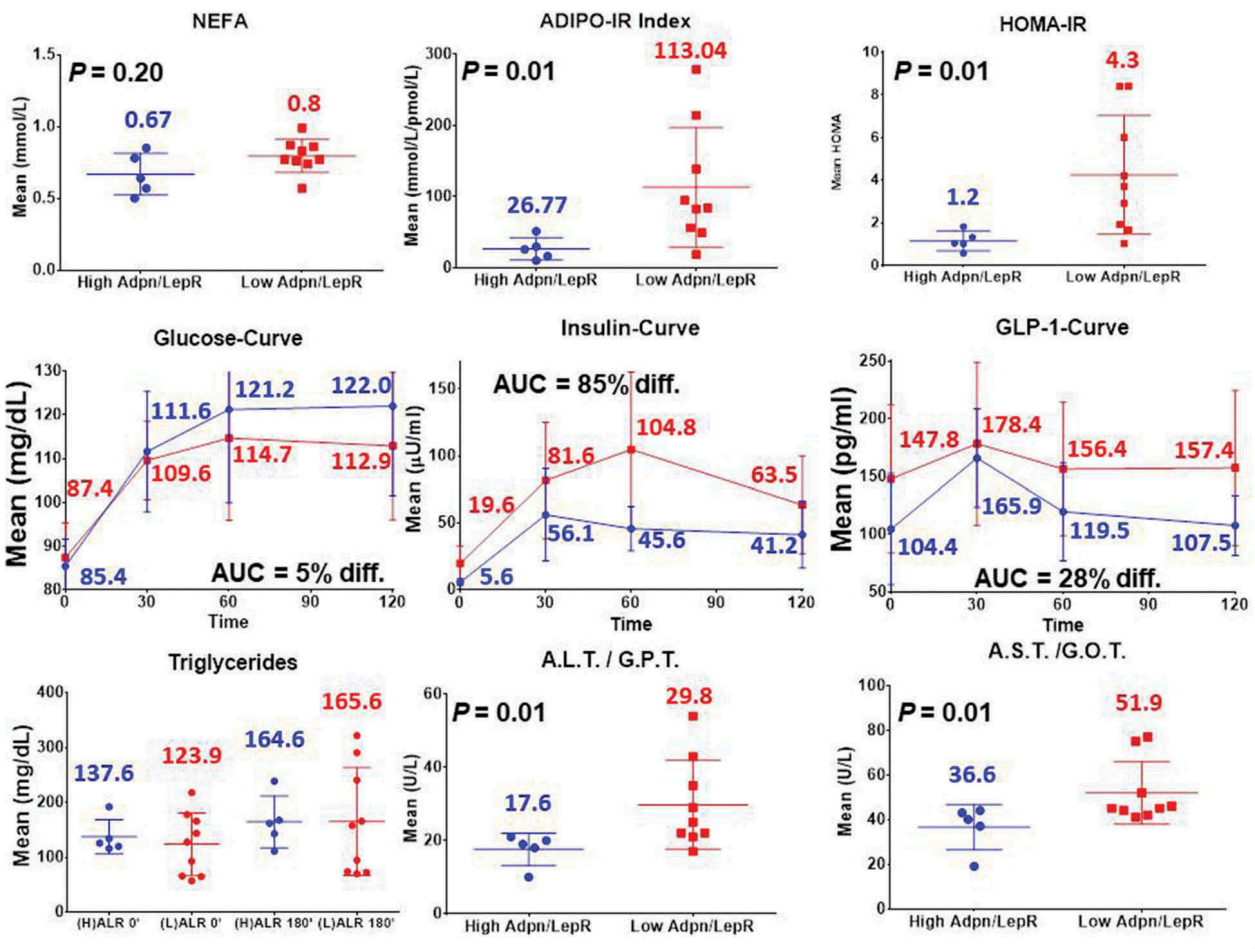
Immunometabolic, insulin resistance, liver enzyme profile, postprandial insulin-glucose axis and triglyceride curves of female participants in the GEMM study (n = 14). Adipo-IR Index, HOMA-IR, ALT/GPT and AST/GOT with a *P-*value ≤ 0.05. Area under the curve (AUC) for glucose in the (h) ALR group and (l) ALG (13,743 and 13,144, 5% difference [diff.]). AUC for insulin and GLP-1 in the (H) ALR group (5055 and 15,143) compared to the (L) ALG (9362 and 19,327) respectively, showed an 85% and 28% diff

Fasting and postprandial insulin-glucose axis phenotypes showed an increase in the (L)ALR subjects (Figure 4) for HOMA (*p* = 0.01), insulin and GLP-1 curves, except for the glucose curve. The area under the curve (AUC) for glucose in the (H) ALR group compared to the (L) ALG showed a 5% difference indicating that they were practically similar. On the contrary, the AUC for insulin and GLP-1 in the (H) ALR group compared to the (L) ALG, respectively, showed an 85% and 28% differences. Systemic lipid metabolism also showed an increase in the (L)ALR symptom-free females for NEFA. The adipose tissue-insulin resistance index (Adipo – IR**_i_**) (*p* = 0.01) was significantly higher in individuals with a (L)ALR. The fasting liver transaminases aspartate transaminase (AST/SGOT) (*p* = 0.01) and alanine transaminase (ALT/SGPT) (*p* = 0.01) were also significantly higher in individuals with a (L)ALR (Figure 4).

For this paper, we decided to present results from fed and fasted samples that underwent shotgun lipidomics only for specific classes considered proinflammatory: Lysophos phatidylethanolamine (LPE), lysophosphatidylcholine (LPC) and Ceramide (Cer) as shown in Table 3. The lipidomic profiling revealed distinct mean differences in plasma lipid composition among the GEMM volunteers. We observed a discrete trend for postprandial differences in a handful of plasma bioactive lipid species at 180 and 300 min on proinflammatory classes of Ceramides (CERN20:0, CERN22:0, CERN23:0), Lyso Phosphatidylethanolamine (LPE18:1), and Lyso Phosphatidylcholine (LPC) (LPC20:3) in the symptom-free (L)ALR compared to the (H)ALR subjects. This is shown in cells highlighted in black from Table 3. The AUC for CERN22:0, LPE18:1 and LPC20:3 in the (H) ALR group compared to the (L) ALG, respectively, showed 8%, 9% and 2.8% differences.

**Table 3.** Multi-dimensional mass spectrometry-based shotgun lipidomics. Fed and fasted shotgun lipidomics data from specific classes considered proinflammatory: Lysophosphatidylethanolamine (LPE), Lysophosphatidylcholine (LPC) and Ceramide (Cer)

	Ceramide(Cer) (nmol/ml plasma)												
Table 3	TIME	A/L R	CERN16:0	CERN18:0	CERN20:0	CERN22:0	CERN23:0	CERN24:2	CERN24:1	CERN24:0	CEROH_N24:1	CEROH_N24:0	
	0 MIN	HIGH	0.34 ± 0.17	0.17 ± 0.07	0.16 ± 0.05	1.00 ± 0.22	0.99 ± 0.28	0.11 ± 0.02	1.57 ± 0.21	3.30 ± 0.95	0.23 ± 0.07	0.09 ± 0.06	
		LOW	0.26 ± 0.06	0.15 ± 0.06	0.15 ± 0.05	1.01 ± 0.37	0.91 ± 0.30	0.11 ± 0.02	1.32 ± 0.37	2.89 ± 0.72	0.18 ± 0.05	0.04 ± 0.02	
	30 MIN	HIGH	0.22 ± 0.05	0.15 ± 0.06	0.11 ± 0.05	0.76 ± 0.31	0.83 ± 0.32	0.09 ± 0.03	1.29 ± 0.37	2.91 ± 1.14	0.18 ± 0.08	0.05 ± 0.02	
		LOW	0.21 ± 0.05	0.13 ± 0.07	0.10 ± 0.06	0.73 ± 0.39	0.72 ± 0.34	0.08 ± 0.03	1.02 ± 0.39	2.31 ± 0.95	0.14 ± 0.05	0.04 ± 0.02	
	180 MIN	HIGH	0.19 ± 0.05	0.10 ± 0.06	0.06 ± 0.04	0.61 ± 0.35	0.71 ± 0.37	0.08 ± 0.05	1.04 ± 0.44	2.32 ± 1.18	0.14 ± 0.07	0.03 ± 0.02	
		LOW	0.22 ± 0.08	0.13 ± 0.07	0.09 ± 0.04	0.71 ± 0.37	0.76 ± 0.33	0.09 ± 0.05	1.06 ± 0.45	2.47 ± 0.88	0.15 ± 0.04	0.04 ± 0.02	
	300 MIN	HIGH	0.22 ± 0.07	0.12 ± 0.04	0.07 ± 0.02	0.60 ± 0.14	0.63 ± 0.16	0.08 ± 0.04	1.13 ± 0.39	2.54 ± 0.65	0.14 ± 0.06	0.06 ± 0.01	
		LOW	0.20 ± 0.06	0.12 ± 0.07	0.10 ± 0.07	0.70 ± 0.31	0.73 ± 0.29	0.09 ± 0.04	1.02 ± 0.40	2.43 ± 0.83	0.15 ± 0.04	0.05 ± 0.04	
Lysophosphatidylethanolamine (LPE) (nmol/ml plasma)													
TIME	A/L R	LPEP16:1	LPEP16:0	LPE16:0	LPEP18:1	LPEP18:0	LPE18:3	LPE18:2	LPE18:1	LPE18:0	LPE20:4	LPE20:3	LPE22:6
0 MIN	HIGH	0.69 ± 0.44	4.87 ± 2.30	9.75 ± 2.73	2.47 ± 0.96	4.47 ± 3.01	0.30 ± 0.10	7.72 ± 2.88	5.60 ± 1.65	11.51 ± 3.22	9.77 ± 5.47	1.00 ± 0.44	2.35 ± 1.15
	LOW	0.57 ± 0.43	3.80 ± 1.27	7.76 ± 2.31	2.27 ± 0.94	3.95 ± 1.33	0.36 ± 0.13	8.12 ± 1.93	5.53 ± 1.53	9.59 ± 1.88	11.52 ± 5.37	1.21 ± 0.40	2.43 ± 1.13
30 MIN	HIGH	0.21 ± 0.12	1.95 ± 0.12	8.85 ± 3.38	0.95 ± 0.46	2.01 ± 1.01	0.30 ± 0.10	7.93 ± 2.56	5.25 ± 1.72	10.44 ± 4.57	7.03 ± 1.74	0.91 ± 0.19	2.16 ± 0.86
	LOW	0.28 ± 0.31	2.04 ± 1.51	6.43 ± 1.87	1.10 ± 0.97	1.88 ± 1.40	0.30 ± 0.14	8.15 ± 2.86	4.89 ± 1.51	7.35 ± 1.65	8.21 ± 4.10	0.97 ± 0.51	1.97 ± 0.79
180 MIN	HIGH	0.18 ± 0.11	2.20 ± 0.69	8.18 ± 1.76	1.06 ± 0.30	2.09 ± 0.41	0.34 ± 0.21	7.15 ± 1.05	5.36 ± 0.63	9.36 ± 2.01	5.02 ± 2.88	0.63 ± 0.26	1.06 ± 0.60
	LOW	0.28 ± 0.90	2.71 ± 1.73	8.56 ± 2.80	1.28 ± 0.98	2.47 ± 1.45	0.43 ± 0.11	10.34 ± 2.64	7.37 ± 1.62	10.07 ± 3.23	6.32 ± 2.88	0.94 ± 0.47	1.38 ± 0.70
300 MIN	HIGH	0.27 ± 0.10	2.30 ± 0.37	10.00 ± 4.65	1.00 ± 0.11	2.06 ± 0.25	0.63 ± 0.39	10.12 ± 4.11	7.08 ± 2.44	10.46 ± 2.90	7.29 ± 4.30	0.95 ± 0.46	1.87 ± 1.36
	LOW	0.22 ± 0.06	3.86 ± 3.90	8.73 ± 6.24	1.63 ± 1.45	3.56 ± 4.03	0.47 ± 0.17	9.54 ± 2.45	7.47 ± 2.00	12.25 ± 10.09	6.36 ± 3.82	0.95 ± 0.55	1.45 ± 0.81
Lyso Phosphatidylcholine (LPC) (nmol/ml plasma)													
TIME	A/L R	LPCP16:0	LPCA16:0	LPC16:1	LPC16:0	LPC18:3	LPC18:2	LPC18:1	LPC18:0	LPC20:4	LPC20:3	LPC20:2	LPC20:1
0 MIN	HIGH	37.12 ± 38.87	3.14 ± 0.82	21.54 ± 15.27	120.54 ± 31. 07	0.76 ± 0.18	42.41 ± 12.88	24.43 ± 5.83	27.07 ± 7.00	9.28 ± 2.61	2.99 ± 0.63	0.93 ± 0.39	0.52 ± 0.15
	LOW	22.23 ± 15.58	2.50 ± 0.38	18.24 ± 9.30	96.82 ± 11.93	0.86 ± 0.36	39.87 ± 10.54	20.40 ± 5.39	21.63 ± 3.43	7.14 ± 1.91	2.69 ± 0.69	1.08 ± 0.40	0.70 ± 0.79
30 MIN	HIGH	4.23 ± 5.18	2.55 ± 0.66	6.20 ± 3.38	114.05 ± 32.40	0.83 ± 0.22	45.16 ± 15.94	23.90 ± 6.06	25.01 ± 7.90	9.46 ± 3.26	3.07 ± 0.92	1.32 ± 0.33	0.58 ± 0.09
	LOW	8.36 ± 21.27	2.12 ± 0.51	8.55 ± 13.11	87.32 ± 12.58	0.78 ± 0.30	39.14 ± 10.90	18.94 ± 5.82	18.70 ± 3.15	6.64 ± 1.67	2.67 ± 1.05	1.29 ± 0.61	0.47 ± 0.13
180 MIN	HIGH	1.35 ± 0.94	2.58 ± 0.70	5.21 ± 2.16	117.18 ± 27.66	0.90 ± 0.28	48.06 ± 13.71	22.34 ± 5.15	23.34 ± 4.99	9.24 ± 3.33	2.94 ± 1.23	0.98 ± 0.78	0.53 ± 0.35
	LOW	2.59 ± 3.34	2.42 ± 0.63	5.53 ± 2.73	107.69 ± 26.04	0.99 ± 0.43	53.03 ± 16.75	22.87 ± 8.54	22.36 ± 5.25	7.56 ± 1.79	2.99 ± 1.36	1.23 ± 0.57	0.52 ± 0.17
300 MIN	HIGH	2.49 ± 3.54	2.34 ± 0.76	4.93 ± 2.87	106.19 ± 38.14	0.91 ± 0.33	52.80 ± 22.19	23.03 ± 8.09	22.13 ± 7.34	9.50 ± 3.72	2.99 ± 1.12	0.95 ± 0.35	0.53 ± 0.19
	LOW	2.49 ± 3.12	2.46 ± 1.39	5.80 ± 3.59	113.44 ± 71.40	0.97 ± 0.47	52.19 ± 16.15	23.00 ± 9.46	24.93 ± 17.73	8.29 ± 2.75	3.18 ± 1.71	0.93 ± 0.60	0.43 ± 0.20

Comprehensive fed/fasted circulating miRNA profiling was performed in this initial sample. We detected 2974 miRNAs as described in the methods. Table 4 shows the results for the most relevant miRNAs signatures we found. They were selected from recent literature on the role of microRNAs in dysfunctional adipose tissue [20], cardiometabolic disorders [32] and the immune response [33]. Our mean values from subjects with a (L)ALR for 0 and 180 min for miRNA promoting adipogenesis showed an increment for miR-27b-5p, miR-378a-3p, miR-375 and miR-140-5p (expressed only in postprandium). Values for miRNA promoting anti-adipogenesis also showed increases for miR-33a-5p, miR-130b-3p, miR-7-1-3p, let-7a-3p (expressed only in postprandium).

**Table 4.** Plasma MicroRNA (miR) in symptom-free females with (H) and (L) ALR. Fed and fasted circulating miRNAs selected from recent literature with adipogenesis-promoting or anti-adipogenic function. miR-27b-5p (fasting and postprandial), miR-375 (postprandial), miR-140-5p (postprandial), miR-130b-3p (fasting and postprandial) with a *P-*value ≤ 0.05

		Adiponectin/leptin ratio					
Table 4		TIME 0 (Fasting)			TIME 180 (Postprandial)		
FUNCTION	miRNA	HIGH	LOW	*P*	HIGH	LOW	*P*
ADIPOGENESIS PROMOTING miRNA	miR-27b-5p	41.2 ± 26.7	65.1 ± 14.6	0.02	41.3 ± 26.6	64.3 ± 16.0	0.04
	miR-378a-3p	54.5 ± 39.9	86.7 ± 15.5	0.11	52.7 ± 37.8	86.1 ± 11.1	0.11
	miR-375	18.1 ± 12.2	35.0 ± 16.9	0.08	17.9 ± 11.3	41.7 ± 21.3	0.01
	miR-140-5p	/	/	/	11.0 ± 6.4	16.7 ± 2.1	0.02
ANTI-ADIPOGENIC miRNA	miR-33a-5p	17.5 ± 8.3	28.4 ± 15.0	0.23	18.8 ± 6.6	27.4 ± 15.0	0.11
	miR-130b-3p	24.8 ± 16.3	41.2 ± 8.0	0.01	23.6 ± 15.7	48.1 ± 8.5	0.02
	miR-7-1-3p	0.006 ± 0.01	0.4 ± 0.5	0.06	0.005 ± 0.01	0.4 ± 0.5	0.06
	let-7a-3p	/	/	/	20.9 ± 14.3	35.0 ± 15.3	0.08

**Figure 5.**
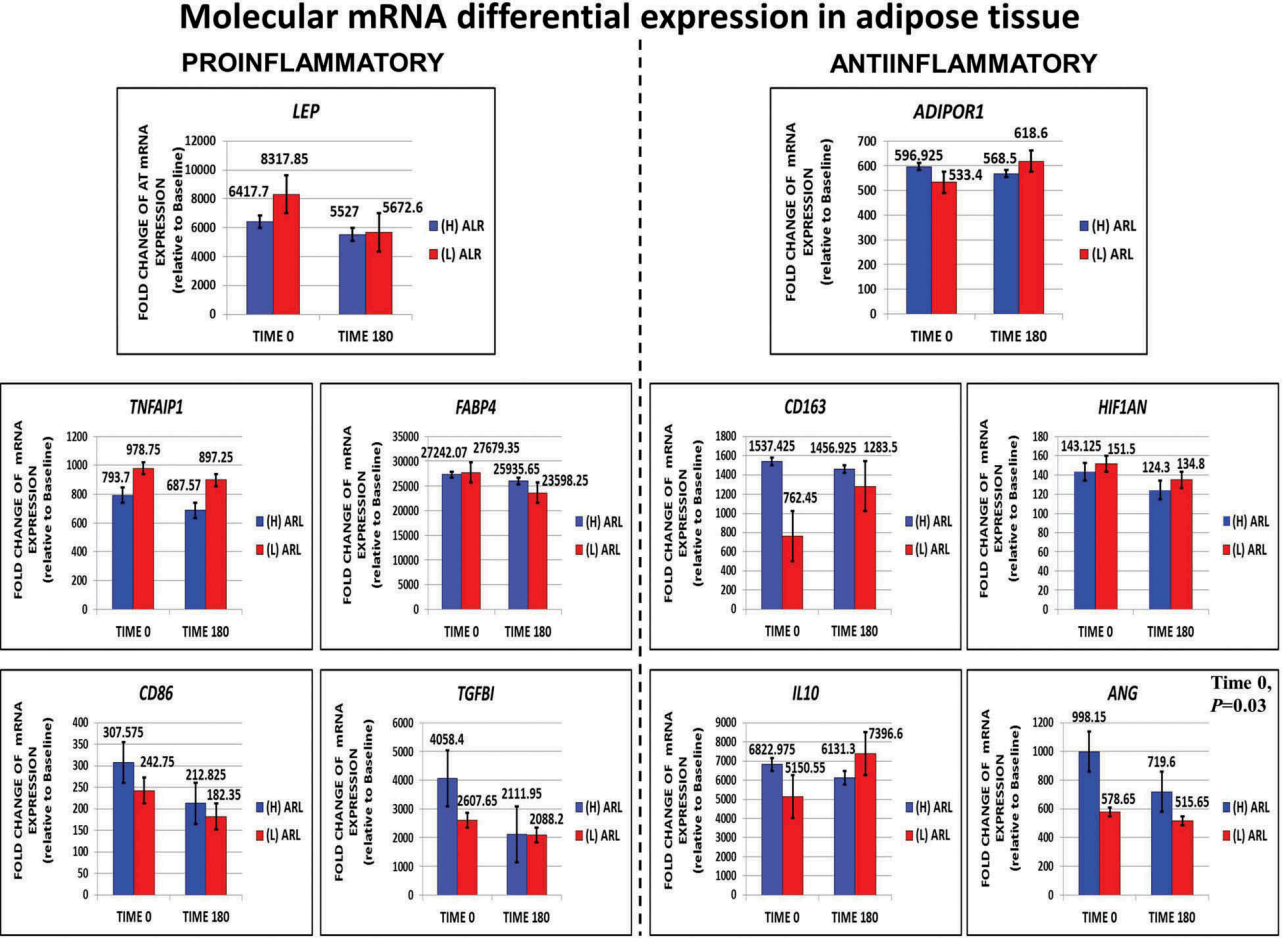
Direct measurements performed for fasting (0ʹ) and fed (180ʹ) subcutaneous fat transcriptomic profiling looking for molecular trends in adipose tissue dysfunction or inflammation differential gene expression in key proinflammatory and anti-inflammatory genes. The expression of the anti-inflammatory gene *ANG* was significantly decreased in the (l)ALR subgroup in fasting

We also quantified mRNA levels of F/P subcutaneous adipose tissue for whole genome expression analysis. We were able to directly measure and characterize key proinflammatory (*LEP, TNFaIP1, CD86, FABP4, TGFB1)* and anti-inflammatory genes (*ADIPOR1, CD163, HIF1AN, IL10, ANG)* at 0 min (fasted) and 180 min (fed). Values are shown in Figure 5. Most genes in the fasted and fed state did not reach statistical significance, except for *ANG (P *= 0.03).

## Discussion

There is a current lack of clinically oriented indicators to assess the complex phenomenon of AT dysfunction for early detection of cardiovascular and immunometabolic risk before it develops into an evident systemic (muscle and liver) IR and an overt metabolic syndrome. It has been stated that the body mass index (BMI) [34], waist circumference (WC) [35], MRI and CT imaging [36] are not ideal predictors of mortality risk or cardiovascular risk factors due to inaccuracies reflecting body fat percentage. But a deeper analysis reveals that they all have the same common approach: they only ascertain for the amount or excess of body fat accumulation in correlation to cardiometabolic risk without taking into account adipose tissue appropriate physiologic function. Moreover, they do not reflect any metabolic or immune feature at the molecular level regarding dysfunctional adipose tissue biology [37]. Therefore, a useful and accurate systemic biomarker to reflect cellular AT dysfunction as an early predictor of cardiovascular and immunometabolic risk is strongly needed.

Using deep phenotyping [38] such as the ALR, the HOMA-IR (from measurements of fasting plasma glucose and insulin concentrations primarily reﬂecting hepatic insulin resistance [39]), the Adipo-IRi and postprandial metabolism curves of the insulin-glucose axis as anchors, we performed precision medicine screening approaches such as F/P plasma microRNA signatures, multi-dimensional mass spectrometry-based shotgun F/P lipidomics, comprehensive plasma metabolic profiling of chronic low-grade subclinical inflammation markers [31] and F/P adipose tissue gene differential expression (transcriptomics) among a selected subgroup of female adult volunteers from the GEMM Family study to identify early trends of cardiovascular and immunometabolic risks (Figures 1 and 2, and Table 2) [40].

A pattern of increased circulating leptin concentrations along with a decreased levels of adiponectin seems indicative of an impaired adipose tissue adipokinome, as it was found in the subgroup with a (L)ALR (Figure 2) [41]. As stated earlier, the adiponectin/leptin ratio (ALR) has been suggested as a marker of adipose tissue (AT) dysfunction [42]. Data in Figure 4 show that the mean HOMA was frankly elevated and the insulin and GLP-1 postprandial curves and their AUC were increased in the (L)ALR group compared to the females with a (H)ALR. Particularly, the insulin curve shows a striking elevation in the females with (L)ALR.

These constant elevated postprandial levels of insulin in the symptom-free females with a (L)ALR may explain why the postprandial glucose curve and the AUC results in the same group were normal, corroborating that in nondiabetic individuals, the β-cells can compensate for resistance to insulin-mediated glucose disposal and maintain normoglycemia at the expense of increased levels of insulin that, unfortunately, is prothrombotic [43]. These frequent postprandial daily peaks of insulin may induce atherothrombotic mechanisms, reducing fibrinolytic balance, and impairing endothelial function as it has been shown using the pancreatic clamp technique in humans [44]. It should be kept in mind that these volunteer subjects had their postprandial metabolic response after a mixed meal challenge with a balanced macronutrient composition (65% carbohydrate, 15% protein and 20% fat) corresponding to 30% of their total daily energy expenditure after a 12-hour fasting [18].

The appearance of deleterious cardiovascular and immunometabolic risk phenotypes has been the main concern regarding body fat accumulation [14]. However, in certain individuals, the more they accumulate an excess of body fat, the less they develop cardiometabolic disease. They can be considered metabolically healthy despite their high degree of body fat accumulation and their long-standing obesity. This effect can be thought of as healthy AT expansion. On the other hand, unhealthy AT expansion is a major contributor to the systemic metabolic disturbances that are characteristic of obesity and type 2 diabetes [45]. The loss of expansion capacity can occur in patients with normal weight, explaining the existence of metabolically unhealthy lean subjects [46]. As a premature event in unhealthy AT expansion, hypoxia likely plays a fundamental role in the initiation of inflammation, leading adipocytes to release proinflammatory factors such as TNF-α, IL-6, hsCRP, PAI-1 and MCP-1. Ultimately, inflammation and AT dysfunction ensues and IR develops, leading to early risk for prediabetes [47].

Metabolically driven inflammation is a hallmark of CVD and T2D [5]. TNF-α, produced by immune cells, was the first cytokine demonstrated to directly impede insulin action in the adipocyte [48]. The preliminary data showed higher systemic levels of TNF-α in the females with (L)ALR. IL-6 also produced by inflammatory cells has been shown to inhibit insulin signalling in the adipocyte as well [49]. Circulating levels of IL-6 were elevated among the symptom-free volunteers with (L)ALR. This ALR has also been correlated with markers of low-grade chronic inﬂammation, such as CRP [8]. CRP has emerged as one of the best predictors of vascular inﬂammation, metabolic syndrome and CVD. The link between low-grade chronic subclinical inflammation, hypoxia and adipocyte dysfunction is the release of cytokines mainly TNF-α and IL-6 into the circulation by adipose tissue, stimulating hepatic CRP production [50]. Our results showed a clear trend of elevated systemic hs-CRP among subjects with (L)ALR. AT dysfunction is characterized by an increased secretion of plasminogen activator inhibitor (PAI)-1 contributing to impair the fibrinolytic system. It seems that this is the link between dysfunctional AT and endothelial damage, platelet reactivity, enhanced coagulation and impaired fibrinolysis, mechanisms currently recognized to increase arterial thrombotic risk [43]. The pattern for circulating levels of PAI-1 in the subgroup with (L)ALR showed a marked elevation. We also measure circulating levels of IL-10. This is a Th2-type cytokine that inhibits the synthesis and activity of proinﬂammatory cytokines and counteracts Toll-like receptor-mediated inﬂammation. IL-10 seems to attenuate obesity-mediated inﬂammation and improve insulin sensitivity in skeletal muscle [51]. These trends of key AT dysfunction immunometabolic phenotypes (an increase in TNF-α, IL-6, hs-CRP, PAI-1 and a decrease in IL-10) mirroring subclinical systemic metaflammation, found in the symptom-free cohort with a (L)ALR, are shown in Figure 3.

A consequence of chronic positive energy balance leading to AT dysfunction is an ectopic deposition of NEFA as triacylglycerols in the liver, skeletal muscle, and pancreas promoting lipotoxicity [52]. Adipose tissue affects triglyceride metabolism by releasing free fatty acids into the circulation, contributing to insulin resistance and eventually leading to abnormalities in lipid metabolism and hypertriglyceridaemia [53]. A validated adipose tissue-insulin resistance (IRi) index (Adipo-IR**_i_** = plasma-free fatty acids (NEFA) x fasting plasma insulin [FPI] [mmol/L/pmol/L]) is calculated based on the linear relationship between the rise in the FPI level and inhibition of the rate of fasting plasma NEFA [54]. The higher the rate of fasting plasma NEFA levels, the greater the severity of adipose tissue IR [55]. We found a triglyceride curve, NEFA levels and an Adipo-IR**_i_** markedly elevated in the (L)LAR subjects when compared to the (H)LAR ones (Figure 4). Liver enzyme levels were also elevated in the (L)ALR participants.

The lipidome is a complete set of lipid species existing in a cell, an organ, or a biological system. Lipidomics has become one of the most important branches of omics [56]. The lipidomic profiling (Table 3) revealed distinct differences in plasma lipid composition among the GEMM volunteers. We observed a discrete trend for a postprandial increase in some plasma bioactive lipid species at 180 and 300 min on proinflammatory Ceramides (CERN20:0, CERN22:0, CERN23:0), Lyso Phosphatidylethanolamine (LPE18:1), and Lyso Phosphatidylcholine (LPC) (LPC20:3) classes in the symptom-free (L)ALR subjects. Of note, the multi-dimensional mass spectrometry-based shotgun lipidomics technique [57] has been widely used to identify altered lipid metabolism and biomarkers under pathophysiological conditions such as prediabetics and type 2 diabetics compared to otherwise healthy subjects [58]. Here we are presenting data on the normal variation among otherwise symptom-free individuals.

MicroRNAs (MiR) have earned great deal of attention not only for their ability to regulate adipogenesis and adipose function, but also for their presence in circulating blood leading to potential tools as diagnostic biomarkers [20]. Table 4 shows the results for the most relevant miRNAs observed, when comparing symptom-free individuals with a (H)ALP vs. a (L)ALP in this cohort of 14 females. miR-27b-5p, miR-378a-3p and miR-375 showed a steady increase in both fasting and postprandial states in the (L)ALR participants. Over-expression of miR-27 results in robust and specific inhibition of adipogenic differentiation with the blockade of *PPARγ* and *C/EBPα* expression [59]. Mir-378a-3p induces adipogenesis by targeting mitogen-activated protein kinase 1 (MAPK1) [60]. miR-375 as an important modulator of β-cell functions. miR-375 overexpression in β-cells leads to a reduction of the number and viability of β-cells [61]. miR-140-5p was only expressed in the fed state and showed an increase in the (L)ALR participants. It participates in modulating the expression of proangiogenic factors, influencing inflammatory reactions [62]. miR-33a-5p and miR-7-1-3p also showed a steady increase in both fasting and postprandial states in the (L)ALR participants. miR-33 is associated with adipose tissue differentiation and development of gastrointestinal tract [63]. Lower expression levels of miR-130 have been reported in the abdominal subcutaneous adipose tissue and in the plasma of obese women compared with those of lean subjects [64]. The let-7 miRNA family plays a key role in modulating inflammatory responses. Recent research has documented that let-7 levels are decreased in diabetic human carotid plaques [65].

Temporal gene expression changes during the fasted and fed state in proinflammatory *LEP, TNFAIP1, CD86, RBP4 and TGFβ* and anti-inflammatory *ADIPOR1, CD163, IL10, HIF1AN* and *ANG* activity to directly measure the balance of the inflammatory/anti-inflammatory response during the development of cellular adaptive responses in early adipose tissue expansion and remodelling have not been fully elucidated [66]. We measured metabolic and immune molecular gene expression directly as a means to compare differential expression features of early trends for adipose tissue dysfunction (Figure 5). We detected a pattern of expression for proinflamatory genes. *LEP* and *TNFAIP1* increased in the (L)ALR subjects. In activated macrophages, proinflammatory M1 and M2b are the main cell types expressing and secreting TNF‐α [67]. Unexpectedly, *CD86* gene expression was decreased in subjects with (L)ALR. Several studies have shown that CD86 is expressed in and used as a marker to identify M1 pro-inflammatory and polarized macrophages [68]. Retinol binding protein 4 (*RBP4*) is secreted by adipocytes, is increased in obese and insulin resistant subjects and induces proinflammatory cytokines through the JNK and TLR-4 pathways in macrophages [69]. *RBP4* did not show any apparent change in expression in either (H) or (L)ALR participants. *TGFβ* is a master regulator and promoter of fibrosis in adipose tissue [70]. Elevated levels of *TGFβ* expression were found in fasting in (H)ALR participants, decreasing in the fed state. *TGFβ* can also be anti-inflammatory and promote M2-like macrophage activity that localize to fibrotic areas of adipose tissue [66].

We also characterized the pattern of expression for AT anti-inflammatory genes (Figure 5). The *ADIPOR1* receptor expression showed a slight increase in the postprandial (L)ALR group. This and several other papers [71] have shown that circulating chronic subclinical inflammation markers are significantly associated with a systemic decrease in adiponectin concentrations in individuals with a (L)ALR leading to insulin resistance [8]. In addition, there is a deleterious decline in AdipoR1/R2 mRNA expression leading to a decrement in adiponectin binding to cell membrane which deeply attenuates the effects of adiponectin [72]. A modest increase was also noted in *CD163* and *IL10* postprandial gene expression in the same group. Macrophages with *CD163* expression are considered anti-inflammatory M2 macrophages [73]. *IL-10* is decreased in subjects with impaired glucose tolerance and obesity [74]. This reduction plays a key role in inflammation-mediated macrophage polarization observed in adipose tissue dysfunction [75]. *ANG* is an important group of vascular remodelling angiogenic factors expressed in adipose tissue that control vessel maturation, patterning, and stabilization [76]. Local hypoxia is a potent stimulus for new blood vessel formation through the pro-angiogenic actions of HIF-1α and HIF-2α [77]. We found a marked decrease of *ANG* in the postprandial (H)ALR subjects with apparently no change in the (L)ALR group.

Some potential limitations of this study should be pointed out. First, due to the small number of subjects, these findings should be interpreted with caution and considered as hypotheses generating. These results should be confirmed by studies with a larger number of subjects. Second, as we used the ALR [9] to compare the immunometabolic systemic and molecular adipose tissue phenotypes, and only total adiponectin was measured, it would be interesting to compare the adiponectin/leptin ratio with the measured fasting and postprandial phenotypes if high-molecular-weight adiponectin was used instead of total adiponectin. On the other hand, leptin and adiponectin are very stable in plasma or serum. As this study included participants who had samples taken in the fed state, this could be an advantage because removing the need for fasting samples would significantly increase the efficiency and feasibility of early immunometabolic and cardiovascular risk measurements in large population-based studies. Third, this study was conducted with a Mexican-mestizo population, therefore it would need to be determined whether these ﬁndings extend to other ethnic groups. However, Mexicans share with Mexican Americans an elevated risk of CVD and T2D [78]. This shared, elevated prevalence of CVRIMO suggests shared genetic factors [79]. As the source population, Mexico reflects the allelic diversity resulting from the conquest and subsequent confluence of European and Native American origins, and therefore reflects the full extent of the spectrum of risk [80]. Finally, we did not adjust for the female’s menstrual cycle stage. Notwithstanding these limitations, this study affirms the central role of adipose tissue dysfunction in triggering the accumulation of predominantly pro-inflammatory immune cells that act as a potent stimulus towards the immunometabolic dysfunction of this tissue leading to adipocyte hypertrophy, fibrosis and hypoxia, which activates macrophage infiltration that ultimately results in insulin resistance.

We are beginning to understand the importance of the complex interactions between inflammation, the extracellular matrix (ECM), and angiogenesis in the context of AT dysfunction. Indeed, our study was carried out in a group of symptom-free normoglycemic, mainly normal-weight women with no history of age-related chronic diseases associated with immunometabolic abnormalities. Moreover, as IR can precede the dysglycemic states of prediabetes and type 2 diabetes mellitus (T2DM) by a number of years and is an early marker of risk for immunometabolic and cardiovascular disease, our early research findings raise several important questions: Does the ALR predict early cardiometabolic risk? Does the elevation of the inflammatory markers (within the normal range) in these symptom-free individuals relate to early risk for endothelial dysfunction and cardiovascular disease? Do the postprandial insulin-glucose axis abnormalities, HOMA-IR and adipo-IRi elevations reflect early risk for prediabetes? Could the correlations of systemic lipid species and microRNAs along with the direct molecular characterization of adipose tissue immunometabolic function lead to early biomarkers of risk for metabolic and cardiovascular disease before the development of frank insulin resistance? It is expected that concrete answers to this question will pave the way for the identification of novel biomarkers to diagnose adipose tissue dysfunction perhaps without the need to fully account for adipose tissue accumulation in a subgroup of symptom-free individuals.

A valid take-home message relates to the 1988 American Diabetes Association Banting award lecture [81], where the late Professor Emeritus of Medicine Gerald ‘Jerry’ Reaven, MD, introduced the concept of the link between IR and a constellation of lipid and non-lipid risk factors of metabolic origin (increased blood pressure, high blood sugar and abnormal HDL cholesterol and triglyceride levels) [82]. This cluster later became known as Metabolic Syndrome [83]. The main messages this outstanding scientist and educator left for the diabetes scientific community stated that values for insulin-mediated glucose disposal vary continuously throughout a population of apparently healthy individuals, with at least a sixfold variation between the most insulin sensitive and most insulin resistant of these individuals, and that approximately one-third of an apparently healthy population is sufficient insulin resistant to develop significant clinical disease [84]. He brilliantly concluded that the primary value of the concept of insulin resistance is that it provides a conceptual framework with which to place a substantial number of apparently unrelated biological events into a pathophysiologic construct [82]. It must be acknowledged that his pioneering research laid the foundations for the key role that adipose tissue (AT) dysfunction plays in the development of IR. Nowadays, we might be at the dawn to unravel that in apparently symptom-free individuals we could place a cluster of immunometabolic phenotypes related to impaired angiogenesis and hypoxia, inflammation, inappropriate extracellular matrix (ECM) remodelling and macrophage polarization into a systemic and molecular construct coined as adipose tissue dysfunction which triggers the early events leading to the development of insulin resistance.

In conclusion, there is a major demographic and epidemiologic change taking place in the U.S. and worldwide. The preventive approach based on single diseases towards symptom-driven medicine is becoming out-of-date. A precision and personalized medicine linked to the identification of early risk and prevention instead of identification of curative pathological symptoms in immunometabolic and cardiovascular disease is rapidly taking place. Optimism to achieve success is in the horizon due to the overwhelming advancement of genomic medicine, particularly integrative systems biology through multi-OMICS technology and definitions of cardiovascular, metabolic and immune disease risk deep phenotypes for early detection of age-related chronic diseases associated with immunometabolic pathology.
